# Hardware Acceleration-Based Privacy-Aware Authentication Scheme for Internet of Vehicles Using Physical Unclonable Function

**DOI:** 10.3390/s25051629

**Published:** 2025-03-06

**Authors:** Ujunwa Madububa Mbachu, Rabeea Fatima, Ahmed Sherif, Elbert Dockery, Mohamed Mahmoud, Maazen Alsabaan, Kasem Khalil

**Affiliations:** 1 School of Computing Sciences and Computer Engineering, University of Southern Mississippi, Hattiesburg, MS 39406, USA; ujunwa.madububambachu@usm.edu (U.M.M.) rabeea.fatima@usm.edu (R.F.) elbert.dockery@usm.edu (E.D.); 2 Electrical and Computer Engineering Department, Tennessee Technological University, Cookeville, TN 38505, USA; mmahmoud@tntech.edu; 3 Department of Computer Engineering, College of Computer and Information Sciences, King Saud University, Riyadh 11362, Saudi Arabia; malsabaan@ksu.edu.sa; 4 Electrical and Computer Engineering Department, University of Mississippi, Oxford, MS 38677, USA; kmkhalil@olemiss.edu; 5 Department of Electrical Engineering, Assiut University, Assiut 71515, Egypt

**Keywords:** physical unclonable function, Internet of Vehicles (IoV), authentication, autonomous vehicles (AVs)

## Abstract

Due to technological advancement, the advent of smart cities has facilitated the deployment of advanced urban management systems. This integration has been made possible through the Internet of Vehicles (IoV), a foundational technology. By connecting smart cities with vehicles, the IoV enhances the safety and efficiency of transportation. This interconnected system facilitates wireless communication among vehicles, enabling the exchange of crucial traffic information. However, this significant technological advancement also raises concerns regarding privacy for individual users. This paper presents an innovative privacy-preserving authentication scheme focusing on IoV using physical unclonable functions (PUFs). This scheme employs the k-nearest neighbor (KNN) encryption technique, which possesses a multi-multi searching property. The main objective of this scheme is to authenticate autonomous vehicles (AVs) within the IoV framework. An innovative PUF design is applied to generate random keys for our authentication scheme to enhance security. This two-layer security approach protects against various cyber-attacks, including fraudulent identities, man-in-the-middle attacks, and unauthorized access to individual user information. Due to the substantial amount of information that needs to be processed for authentication purposes, our scheme is implemented using hardware acceleration on an Nexys A7-100T FPGA board. Our analysis of privacy and security illustrates the effective accomplishment of specified design goals. Furthermore, the performance analysis reveals that our approach imposes a minimal communication and computational burden and optimally utilizes hardware resources to accomplish design objectives. The results show that the proposed authentication scheme exhibits a non-linear increase in encryption time with a growing AV ID size, starting at 5μs for 100 bits and rising to 39 μs for 800 bits. Also, the result demonstrates a more gradual, linear increase in the search time with a growing AV ID size, starting at less than 1 μs for 100 bits and rising to less than 8 μs for 800 bits. Additionally, for hardware utilization, our scheme uses only 25% from DSP slides and IO pins, 22.2% from BRAM, 5.6% from flip-flops, and 24.3% from LUTs.

## 1. Introduction

In the last few years, progress has been made in smart cities, and information and communication technology has given rise to intelligent urban management and services. In this scenario, the Internet of Vehicles (IoV) plays a crucial role as a fundamental technology linking smart cities with intelligent connected vehicles. It revolutionizes transportation safety and efficiency by establishing wireless communication channels among vehicles. This interconnected network streamlines the exchange of essential traffic-related information, addressing the escalating demand for enhanced transportation safety and efficiency. Consequently, it raises security and privacy concerns among the individual users of these vehicles [1,2,3,4,5,6,7,8,9,10,11].

The challenge of authentication in IoV stems from the heightened connectivity and data exchange among vehicles, giving rise to concerns regarding privacy and security. As vehicles engage in wireless communication, the risk of unauthorized access or malicious attacks on data becomes a significant hurdle, underscoring the importance of ensuring authenticity to preserve the integrity of the IoV ecosystem and protect individual privacy. Additionally, existing privacy-based authentication solutions for IoV encounter limitations, including susceptibility to threats such as data tampering and identity counterfeiting. Conventional approaches may not effectively tackle these challenges, leaving vulnerabilities for potential privacy and security breaches. Moreover, current solutions may not adequately account for the dynamic and open nature of IoV networks, potentially leading to lapses in comprehensive privacy protection [12,13].

Furthermore, existing solutions neglect the authentication of autonomous vehicles (AVs) under various traffic management authorizations or fail to incorporate an additional layer of security to safeguard user privacy. Therefore, the necessary authentication scheme should possess the capability to authenticate AVs under different managements with minimal requirements, introduce additional security features to ensure user privacy and demand minimal resources for implementation. To overcome these limitations, our proposed hardware acceleration-based privacy-preserving authentication scheme for the IoV incorporates the k-nearest neighbor (KNN) encryption technique [14] with a multi-multi searching property and physically unclonable functions (PUFs) [15,16,17,18,19,20,21,22]. This scheme emphasizes privacy-preserving authentication, ensuring a secure and accurate vehicle data exchange. Integrating PUFs in key generation adds an extra layer of security, leveraging unique physical attributes to enhance the overall privacy and security of IoV networks. This scheme emphasizes privacy-preserving authentication, ensuring secure and accurate data exchange among vehicles and traffic management.

This paper presents a symmetric-key searchable encryption scheme for multi-traffic management (such as departments of transportation in various states) and multi-user settings for AVs designed for Internet of Vehicles (IoV) system authentication, wherein data, including AV identification data, are uploaded from various traffic management (TM) and multiple users (AVs). The AV that registers with any traffic management can authenticate itself to any TM. Utilizing current searchable encryption algorithms (intended for single-multi-search settings) for IoV authentication necessitates that each AV possesses a distinct set of keys (one for every TM) and encrypts its identity data utilizing the key for each TM. Consequently, several computation and communication overheads arise, creating a significant challenge in managing these keys. Our authentication scheme permits each AV and TM to utilize a singular key to encrypt the AV identity data vectors (authentication requests for the AV, and identification vectors of all AVs underneath the control of a certain TM) just once while enabling the server to match the encrypted vectors. Moreover, a constraint of current symmetric-key searchable encryption methods [23,24,25] is that anyone who obtains the encrypted vectors, such as via eavesdropping on TM and AV communications, can calculate the similarity score and infer additional information. Our technique effectively addresses this difficulty, necessitating the authentication server (AS) to search through encrypted data utilizing its private key.

Additionally, to address the substantial data volume in IoV applications and potential scalability concerns, our scheme incorporates PUFs for key generation. PUFs are exceptionally challenging to clone and are physically designed to enhance security protocols for AVs. They provide physical security, cost-effectiveness, a simple structure, high throughput with low energy consumption, and unclonable properties. We also implement our authentication scheme by using hardware acceleration on a field-programmable gate array (FPGA) board. Implementing the scheme in VHDL on an FPGA board with varying capabilities showcases its practicality. Our investigation illustrates that our approach efficiently fulfills the required security and privacy design criteria compared to existing schemes. Furthermore, a performance analysis reveals that our approach imposes minimal computational burden and optimally utilizes hardware resources to accomplish design objectives. The results show that the proposed authentication scheme exhibits a non-linear increase in encryption time with a growing AV ID size (security levels different than the ID size will decide the key size), starting at 5 μs for 100 bits and rising to 39 μs for 800 bits. Also, the result demonstrates a more gradual, linear increase in search time, highlighting the efficiency and scalability of the search process even for large ID sizes.

The subsequent sections of this work are organized as follows. The subjects of related works are addressed in Section 2. Section 3 presents a comprehensive overview of our network model, threat model, and design objectives, which are clearly defined. The scheme under consideration is outlined in Section 4. The scheme’s security and privacy analysis can be found in Section 5, while the implementation and performance evaluation are presented in Section 6. In Section 7, conclusive findings are ultimately derived.

## 2. Related Work

Several schemes have been proposed to enhance the security and privacy of the IoV. In [26], the paper concentrates on the security challenges encountered in unmanned aerial vehicle (UAV) networks, offering remedies such as intrusion detection. Additional endeavors encompass enhancing the system’s efficacy, addressing power consumption, and employing image-processing techniques to identify malicious drones. The proposed scheme introduces significant latency and computational overhead, making it less suitable for real-time authentication in dynamic IoV environments. Furthermore, that work does not explore hardware acceleration techniques like our paper. In [27], the authors introduce a scheme to preserve privacy in secure communications between vehicles and infrastructure within vehicular ad hoc networks (VANETs). That paper tackles security challenges, including key escrow and computational overhead, by introducing a certificateless aggregate signature (CLAS) for efficient signature aggregation. Pre-calculation techniques are employed to minimize computational overhead, while the security proof validates the resilience of the scheme against different types of attacks. This scheme relies on certificateless aggregate signatures to improve efficiency, but it assumes that roadside units (RSUs) are semi-trusted, which introduces potential vulnerabilities if compromised.

In [28], the authors focus on using blockchain technology to combat security and privacy threats, such as identity authentication within the IoV. The paper introduces an improved authentication scheme, including a new contract with new nodes, a key distribution scheme based on blockchain and the traditional public key infrastructure authentication, a node joining process, and a node identity authentication process based on blockchain and public key pair technology. However, this scheme incurs high computational overhead, whereas our approach enhances scalability using KNN encryption and PUFs, ensuring efficient authentication with lower resource consumption. Additionally, while their scheme relies on PKI and smart contracts, ours achieves multi-multi searching without requiring multiple keys. In [29], the paper focuses on security and privacy issues within the IoV, such as authentication, cloning attacks, and privacy preservation. The paper proposes a protocol that uses PUFs to provide security and a three-layered infrastructure architecture for IoV to reduce issues such as authentication overhead and improve the throughput of packets within the application layer. However, it does not support multi-multi searching across multiple traffic management entities.

In [30], a new certificateless short signature scheme (CLSS) and an efficient anonymous authentication scheme for IoV are proposed, designed by combining CLSS and a regional management strategy. This scheme lacks scalability for large IoV networks and cross-region authentication. Additionally, its reliance on bilinear pairings introduces computational overhead, making real-time deployment challenging compared to our PUF-based low-latency approach. In [31], the authors introduce novel protocols for safeguarding intellectual property (IP) on FPGAs. They present the development of an intrinsic PUF designed explicitly for FPGAs, analyze the statistical properties of SRAM-based PUFs, explore trade-offs in implementing a fuzzy extractor, and validate the construction of FPGAs with embedded block RAMs. However, this scheme suffers from scalability issues due to Hyperledger Fabric’s permissioned nature and consensus overhead. Additionally, its reliance on RSU-based leader selection introduces potential bottlenecks and delays in high-mobility vehicular networks.

In [32], the authors explore an innovative method for key generation utilizing PUFs. That work introduces a scheme called Positioning Syndrome Coding for extracting keys from groups, facilitating the generation of high-entropy keys with low error correction costs. However, it relies on precise fabrication, making it susceptible to manufacturing variations that can affect reliability. In addition, in [33], the paper centers on a system design that integrates PUFs and channel reciprocity to securely generate and exchange secret keys. This scheme aims to achieve anonymous authentication through which information from legitimate vehicles can be verified and attacks can be prevented. That paper focuses solely on optimizing PUF-based key generation, unlike our hardware-accelerated scheme, which enhances real-time efficiency and scalability. That previous work lacks multi-multi searching capabilities and may introduce processing delays in resource-constrained environments.

In [34], the presented scheme leverages the Hyperledger Fabric blockchain platform to establish a decentralized environment, enabling vehicles to handle their data autonomously during communication and voting procedures. The model employs a three-layered network architecture to diminish authentication overhead while enhancing packet throughput within the application layer. To tackle the leader selection process, the paper introduces a reputation-based scheme. Additionally, it introduces an anonymous yet traceable privacy-preserving and authentication approach tailored to vehicular networks. Its dependency on controlled fading introduces computational complexity, and the approach lacks real-time authentication and scalability for large-scale networks. Additionally, using PUFs for key generation does not address authentication across multiple entities, limiting its applicability to broader IoV or multi-stakeholder systems. In [35], the paper introduced Hardware-Accelerated Authentication (HAA) for securing communication within mission-critical vehicular networks of the Internet of Things, specifically designed for military vehicular networks. The proposed scheme enhances the security and performance of rapid authentication signatures by leveraging graphics processing units (GPUs) to quickly achieve cryptographic protection in high-throughput vehicular networks. Compared to traditional CPU implementations, HAA exhibits superior processing speeds while maintaining an equivalent level of security. The paper’s reliance on GPU acceleration limits deployment in resource-constrained environments. Moreover, it does not elaborate on the secure and scalable distribution of cryptographic keys among dynamic and large vehicular networks.

In [36], the paper proposes CALRA (Conditional Anonymous and Leakage-Resilient Authentication) for vehicular crowdsensing communication (VCS). The paper combines signcryption, bilinear pairings, and identity-based cryptography (IBC) to reduce computational overhead and enhance security. CALRA minimizes cryptographic delays, optimizes ciphertext sizes, and supports fog node authentication for scalability. A trusted authority manages pseudonymous identities, ensuring traceability when needed. CALRA outperforms existing methods by reducing delays, eliminating third-party reliance, lowering energy consumption, and offering stronger resistance to identity forgery and replay attacks. However, its computational complexity still poses limitations in specific scenarios, requiring further evaluation in practical applications.

Unlike previous schemes, our approach addresses the challenge of authenticating vehicles under the control of different management entities. We introduce multi-multi searching properties for AV authentication within the IoV era. Each AV, managed by a specific traffic management entity, no longer needs to share a distinct key with other traffic managemers for authentication. This characteristic significantly enhances the scalability of our scheme. Furthermore, our scheme outperforms previous hardware-based authentication schemes, demonstrating improved efficiency and reduced resource consumption.

## 3. System Model and Design Goals

### 3.1. Network Model

**Figure 1:**
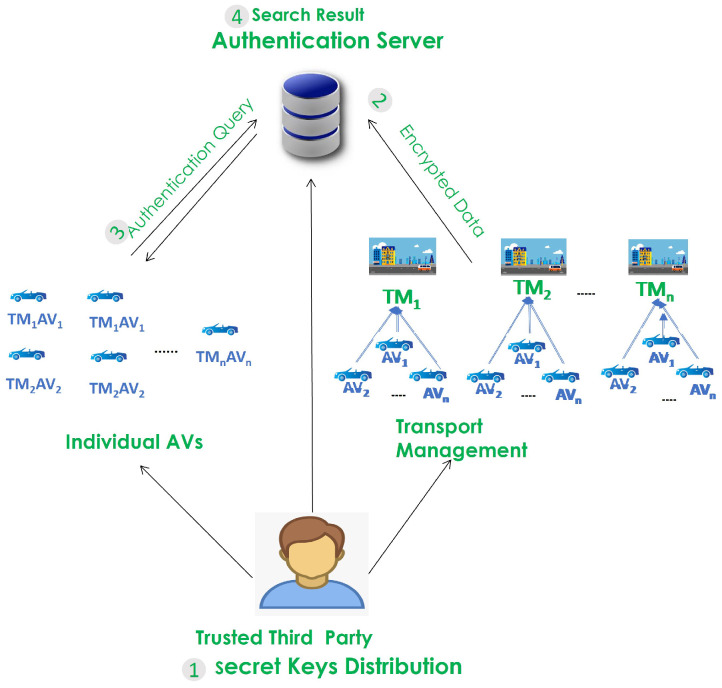
Network model.

The architectural framework of the system comprises four essential components: autonomous vehicles (AVs), transport management entities (TMs), an authentication server (AS), and the trusted third party (TTP), as depicted in Figure 1. The TTP plays a crucial role in distributing a unique secret key generated via a PUF to the AS, each AV, and each TM. Serving as a trusted intermediary, the TTP is universally recognized by all participating entities. Each TM encrypts the AV’s ID, known as indices, associated with all AVs under its control. This encryption utilizes the key provided via the TTP; subsequently, the indices are transmitted to the AS. To achieve self-authentication, each AV encrypts its ID information using the key delivered by the TTP, generating an authentication request commonly referred to as a trapdoor. This encrypted request is then transmitted to the AS. In turn, the AS utilizes the trapdoor to conduct an exhaustive search across all encrypted indices, calculating their similarity scores. Subsequently, it provides an authentication outcome for the AVs.

### 3.2. Threat Model

The threat model covers potential adversaries both internal and external. The internal attack model describes a situation where systems are essentially honest but curious, aiming to gather sensitive information about the entities involved in the scheme, including user information and associated keys. Internal attackers could include the AS, one of the TMs, or AVs. External attackers, on the contrary, take the form of eavesdroppers. The proposed security goals are designed to address and mitigate these threats, primarily to safeguard user privacy within the IoV system.

### 3.3. Design Goals

In light of the outlined threat model above, the design objectives that our proposed scheme should meet are as follows:

- **Scalability and efficiency:** The lightweight and effective execution of encryption and search operations is crucial. This reduces energy and computational costs associated with data access and processing. Simultaneously, it allows authorized individuals to access the required information securely. Moreover, it streamlines the encryption and searching of data with varying volumes and structures, ensuring adaptability to different data sizes and scenarios.
- **Authentication query search over encrypted AVs’ IDs from several TMs:** Our method should be able to utilize the encrypted authentication requests from AVs to explore the encrypted ID data transmitted via various participating TMs. Each AV should use only one key to encrypt its authentication request, and it can still be matched with all indices submitted via all TMs to achieve a multi-multi searching property.
- **AVs’ ID data and authentication query confidentiality:** the AS must refrain from obtaining sensitive or crucial information related to the stored AV’s ID data or the authentication queries/requests.
- **Authentication query unlinkability:** the AS should be unable to ascertain whether two authentication requests include identical or distinct identification data.
- **Distinct key generation:** the TTP must be capable of generating unique keys for distribution to all scheme members, ensuring that they cannot be duplicated or repeated.

## 4. Proposed Authentication Scheme

The proposed scheme comprises the following phases: key generation, key distribution, the encryption of the AV’s identification information, the submission of authentication queries, and the matching of encrypted data and obtaining the authentication results. Table 1 explains our scheme’s main notations.

### 4.1. Key Generation

**Figure 2:**
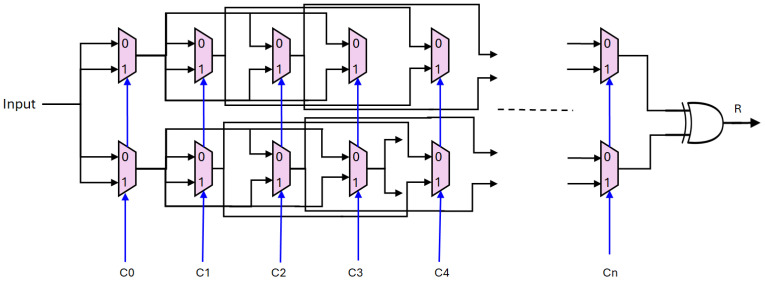
Proposed PUF architecture.

In our proposed authentication scheme, the keys are generated through our proposed PUF architecture. The proposed PUF design is illustrated in Figure 2. It is designed as a sequence of multiplexers arranged in a specific configuration to generate a unique response (R) based on challenge inputs. This PUF architecture consists of multiple stages, each comprising a chain of two multiplexers. In each stage, a challenge input determines the path selection of the input signal towards the output port. Notably, the output of each multiplexer column is not directly connected to the subsequent column but extends its connection to the following two columns in the sequence. This configuration adds complexity and variability to the PUF’s response generation, contributing to its uniqueness and security against duplication attempts.

To elaborate further, let us delve into the operation of each stage within this proposed PUF structure. At the start of the PUF chain, the initial challenge input influences the path selection within the first multiplexer stage, leading to an output signal. This output then branches out to influence the subsequent two multiplexer stages, rather than a direct connection to the immediately following stage. This branching mechanism introduces a non-linear and unpredictable behavior into the PUF’s response generation, enhancing its resistance to modeling attacks and ensuring each PUF instance produces a distinct output for the same challenge input.

The culmination of this PUF architecture is witnessed at the final multiplexer stage, where the output of the last column is fed into an XOR gate to produce the final response. By employing an XOR operation at the endpoint of the PUF chain, the final response is computed based on the combined effects of all preceding multiplexer stages and their respective challenge inputs. This collective integration of challenge-dependent paths and XOR-based computation ensures that the PUF’s response is a function of both the input challenge and the intricate arrangement of multiplexer connections throughout the PUF structure. This design strategy aims to create a reliable and robust PUF that can serve as a secure hardware-based key generation mechanism in various applications requiring intrinsic key uniqueness and resilience against attacks aimed at cloning or emulation.

In the context of the proposed PUF architecture, the challenge inputs $C_{0},C_{1},\ldots ,C_{n}$ play a pivotal role in determining the unique response generated by the PUF for a given operation. Each challenge input $C_{i}$ serves as a control signal that influences the selection of paths through the multiplexer stages. Specifically, at each stage of the PUF chain, the challenge input $C_{i}$ directs the routing of the input signal, dictating which path to take within the multiplexer configuration. This dynamic routing mechanism ensures that even slight variations in the challenge inputs lead to significantly different pathways and distinct responses from the PUF.

The complexity introduced by the sequential application of challenge inputs across multiple stages enhances the entropy and unpredictability of the PUF’s output. As the challenge inputs propagate through the PUF structure, their collective impact on the signal routing creates a highly non-linear relationship between the input challenges and the resulting PUF response. This non-linear behavior, compounded by the branching nature of the multiplexer connections and XOR-based aggregation, contributes to the cryptographic strength of the PUF by thwarting attempts to predict or model its behavior based on observed responses.

Furthermore, the challenge inputs $C_{0},C_{1},\ldots ,C_{n}$ can be considered part of the secret key material for the PUF. The unique combination and sequence of challenge inputs used during PUF operation constitute a secret that is essential for reproducing the correct response from the device. This reliance on challenge inputs as part of the PUF’s operational key ensures that the device’s output remains confidential and resistant to reverse engineering or unauthorized duplication. As a result, the security and effectiveness of the proposed PUF rely not only on its physical design but also on the strategic management and utilization of challenge inputs to harness its inherent unpredictability and cryptographic strength for secure key generation purposes. All keys in Section 4.2 are derived using our proposed PUF design.

### 4.2. Key Distribution

- **System Setup:** The system setup algorithm takes the security parameter $1^{n}$ as an input and outputs the TTP secret key (TTPK), where TTPK = {*B*, $P_{1}$, $P_{2}$, $M_{1}$, …, and $M_{8}$}, *B* is a random binary vector of size n, and $P_{1}$, $P_{2}$, $M_{1}$, ……, and $M_{8}$ are n*n invertible random matrices, and n is the size of the AV identification size.
- **KeyGenCloud:** The TTP creates the authentication server secret key (ASK), where ASK = A, A is an invertible random matrix $\in R^{n\times n}$.
- **KeyGenTM:** For transport management *i* with ID ($TM_{i}$), the TTP creates its secret key ($TMK_{i}$) as $TMK_{i}$ = {*B*, $AM_{1}^{-1}C_{i}$, $AM_{2}^{-1}D_{i}$, $AM_{3}^{-1}C_{i}$, $AM_{4}^{-1}D_{i}$, $AM_{5}^{-1}E_{i}$, $AM_{6}^{-1}F_{i}$, $AM_{7}^{-1}E_{i}$, $AM_{8}^{-1}F_{i}$}, where {$C_{i}$, $D_{i}$, $E_{i}$, and $F_{i}$} are random matrices $\in R^{n\times n}$, such that $C_{i}$ + $D_{i}$ = $P_{1}^{-1}$, and $E_{i}$ + $F_{i}$ = $P_{2}^{-1}$.
- **KeyGenAV:** For autonomous vehicle *x* with ID ($AV_{x}$), the TTP creates its secret key ($AVK_{x}$) as $AVK_{x}$ ={*B*, $H_{x}M_{1}$, $H_{x}M_{2}$, $J_{x}M_{3}$, $J_{x}M_{4}$, $K_{x}M_{5}$, $K_{x}M_{6}$, $N_{x}M_{7}$, $N_{x}M_{8}$}, where {$H_{x}$, $J_{x}$, $K_{x}$, and $N_{x}$} are random matrices $\in R^{n\times n}$, such that $H_{x}$ + $J_{x}$= $P_{1}$, and $K_{x}$ + $N_{x}$ = $P_{2}$.

### 4.3. Encrypting an AV’s ID Data

Each $TM_{i}$ would use vector *B* to split its data vector ($q_{i}$), every AV’s ID data, into two random column vectors, $q_{i}^{\prime }$ and $q_{i}^{\prime \prime }$, of the same size, n. For each bit $q_{i}\left(j\right)$, if B(j) is 1, $q_{i}^{\prime }\left(j\right)$ and $q_{i}^{\prime \prime }\left(j\right)$ are set similarly to $q_{i}\left(j\right)$; meanwhile, if B(j) is 0, $q_{i}^{\prime }\left(j\right)$ and $q_{i}^{\prime \prime }\left(j\right)$ are set to two random numbers, such that their summation is equal to $q_{i}\left(j\right)$.

Then, $TM_{i}$ uses its encryption key, $TMK_{i}$, to encrypt the vector pair ($q_{i}^{\prime },q_{i}^{\prime \prime }$) and generate the ciphertext $S_{ix}$ for $AV_{x}$ under the control of $TM_{i}$, called the index. $S_{ix}$ is a column vector of size 8n, and it will transmit $S_{ix}$ to the AS. $S_{ix}$ = [$AM_{1}^{-1}C_{i}q_{i}^{\prime }$, $AM_{2}^{-1}D_{i}q_{i}^{\prime }$, $AM_{3}^{-1}C_{i}q_{i}^{\prime }$, $AM_{4}^{-1}D_{i}q_{i}^{\prime }$, $AM_{5}^{-1}E_{i}q_{i}^{\prime \prime }$, $AM_{6}^{-1}F_{i}q_{i}^{\prime \prime }$, $AM_{7}^{-1}E_{i}q_{i}^{\prime \prime }$, $AM_{8}^{-1}F_{i}q_{i}^{\prime \prime }$].

### 4.4. Submitting Authentication Query

Each individual AV uses its key received from the TTP to encrypt the authentication query and sends it to the AS. To create an encrypted authentication query, each individual $AV_{x}$ uses part of its secret key (B) received from the TTP as a splitting indicator to split its ID information vector ($U_{x}$) into two random vectors, $u_{x}^{\prime }$ and $u_{x}^{\prime \prime }$. If the B(y) bit is zero, both $u_{x}^{\prime }\left(y\right)$ and $u_{x}^{\prime \prime }\left(y\right)$ are set similarly to U(y). Conversely, if the B(y) is one, $u_{x}^{\prime }\left(y\right)$ and $u_{x}^{\prime \prime }\left(y\right)$ are assigned random values, ensuring that their combined sum equals U(y). Subsequently, $AV_{x}$ employs its designated secret key, represented as $AVK_{x}$, to create the trapdoor, referred to as ($CT_{x}$). $CT_{x}$ = [$u_{x}^{\prime }H_{x}M_{1}$, $u_{x}^{\prime }H_{x}M_{2}$, $u_{x}^{\prime }J_{x}M_{3}$, $u_{x}^{\prime }J_{x}M_{4}$, $u_{x}^{\prime \prime }K_{x}M_{5}$, $u_{x}^{\prime \prime }K_{x}M_{6}$, $u_{x}^{\prime \prime }N_{x}M_{7}$, $u_{x}^{\prime \prime }N_{x}M_{8}$]. $CT_{x}$ is a row vector of size 8n. Then, the $AV_{x}$ transmits $CT_{x}$ to the AS for authentication.

### 4.5. Matching Encrypted Data

The AS will utilize the KNN encryption scheme to perform data searches without needing to decrypt the data. Subsequently, the AS will employ the search result to authenticate the AV user’s query. The AS should first use its secret $A^{-1}$ to remove A from $S_{ix}$ in order to obtain $S_{ix}^{\prime }$.

$S_{ix}^{\prime }$ = [$M_{1}^{-1}C_{i}q_{i}^{\prime }$, $M_{2}^{-1}D_{i}q_{i}^{\prime }$, $M_{3}^{-1}C_{i}q_{i}^{\prime }$, $M_{4}^{-1}D_{i}q_{i}^{\prime }$, $M_{5}^{-1}E_{i}q_{i}^{\prime \prime }$, $M_{6}^{-1}F_{i}q_{i}^{\prime \prime }$, $M_{7}^{-1}E_{i}q_{i}^{\prime \prime }$, $M_{8}^{-1}F_{i}q_{i}^{\prime \prime }$].

Then, $CT_{x}$ * $S_{ix}^{\prime }$ is performed to prove that the AV is legitimate, where * is a dot product operation. The proof of the above mathematical model is achieved by computing the following.

$CT_{x}$ * $S_{ix}^{\prime }$ = [$u_{x}^{\prime }H_{x}M_{1}$ · $M_{1}^{-1}C_{i}q_{i}^{\prime }$ + $u_{x}^{\prime }H_{x}M_{2}$ · $M_{2}^{-1}D_{i}q_{i}^{\prime }$ + $u_{x}^{\prime }J_{x}M_{3}$ · $M_{3}^{-1}C_{i}q_{i}^{\prime }$ + $u_{x}^{\prime }J_{x}M_{4}$ · $M_{4}^{-1}D_{i}q_{i}^{\prime }$ + $u_{x}^{\prime \prime }K_{x}M_{5}$ · $M_{5}^{-1}E_{i}q_{i}^{\prime \prime }$ + $u_{x}^{\prime \prime }K_{x}M_{6}$ · $M_{6}^{-1}F_{i}q_{i}^{\prime \prime }$ + $u_{x}^{\prime \prime }N_{x}M_{7}$ · $M_{7}^{-1}E_{i}q_{i}^{\prime \prime }$ + $u_{x}^{\prime \prime }N_{x}M_{8}$ · $M_{8}^{-1}F_{i}q_{i}^{\prime \prime }$]

= [$u_{x}^{\prime }H_{x}C_{i}q_{i}^{\prime }$ + $u_{x}^{\prime }H_{x}D_{i}q_{i}^{\prime }$ + $u_{x}^{\prime }J_{x}C_{i}q_{i}^{\prime }$ + $u_{x}^{\prime }J_{x}D_{i}q_{i}^{\prime }$ + $u_{x}^{\prime \prime }K_{x}E_{i}q_{i}^{\prime \prime }$ + $u_{x}^{\prime \prime }K_{x}F_{i}q_{i}^{\prime \prime }$ + $u_{x}^{\prime \prime }N_{x}E_{i}q_{i}^{\prime \prime }$ + $u_{x}^{\prime \prime }N_{x}F_{i}q_{i}^{\prime \prime }$]

= $u_{x}^{\prime }\left(H_{x}+J_{x}\right)\left(C_{i}+D_{i}\right)q_{i}^{\prime }+u_{x}^{\prime \prime }\left(K_{x}+N_{x}\right)\left(E_{i}+F_{i}\right)q_{i}^{\prime \prime }$

= $u_{x}^{\prime }\left(P_{1}.P_{1}^{-1}\right)q_{i}^{\prime }+u_{x}^{\prime \prime }\left(P_{2}.P_{2}^{-1}\right)q_{i}^{\prime \prime }$

= $U_{x}\cdot q_{i}$

If there is a match, the AS accepts the AV user’s data. Otherwise, it rejects the data.

## 5. Security and Privacy Analysis

Based on the design goals outlined in Section 3.3, we demonstrate that our proposed scheme meets all the specified capabilities and criteria by effectively addressing each aim mentioned.

- **Scalability and efficiency.** The proposed method efficiently searches encrypted IDs from multiple sources and promptly responds to requests from the AVs. This is achieved through a dot product operation using the relevant encrypted IDs and leveraging hardware acceleration. Additionally, the computational burden in our technique is relatively manageable.
- **Authentication query search over encrypted AVs’ IDs from several TMs.** The proposed method utilizes encrypted authentication queries sent from an AV to the AS to perform searches on encrypted ID data from various participating TM entities. Our approach eliminates the need for AVs to share a key with each TM for searching its encrypted AVs’ identification data. The AVs can efficiently use the same key obtained from the TTP to search all ID data from all the involved TMs.
- **AVs’ ID data and authentication query confidentiality.** The AS is barred from accessing any potentially sensitive or valuable information regarding stored ID data or issued authentication queries. This is accomplished through encryption via both the AV and TMs. This encryption ensures the confidentiality of IDs and authentication queries, preventing data transmission to the AS in plaintext. Data privacy is heightened, as the server does not decrypt the data, and the AS only conducts searches within the encrypted data.
- **Authentication query unlinkability.** The AS cannot ascertain whether two authentication requests contain similar data. Our technique ensures the preservation of authentication query unlinkability by generating alternative ciphertexts when the same AV ID is sent out through random vector splitting during the authentication query process.
- **Distinct key generation.** Our scheme demonstrates that the PUF generates a unique random key during the process of creating singular keys for distribution, guaranteeing that they are non-duplicable and non-repetitive.

## 6. Implementation and Experimental Results

### 6.1. Hardware Architecture

**Figure 3:**
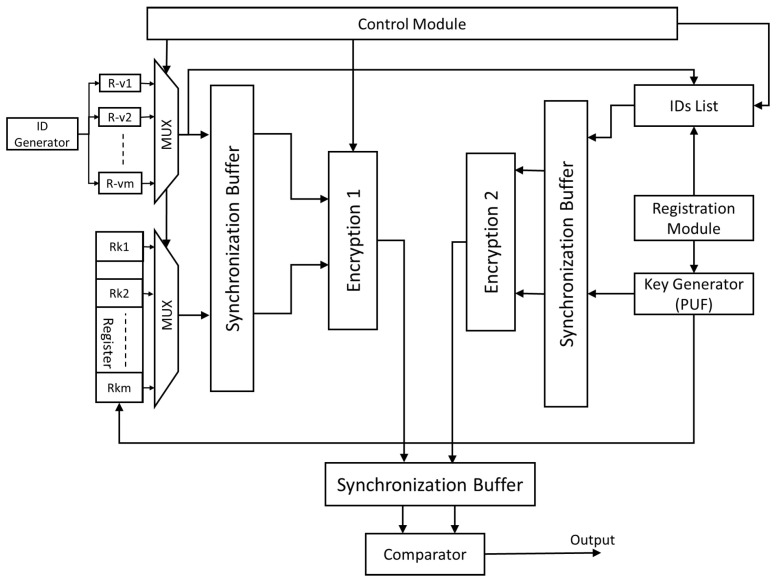
The proposed method architecture.

The proposed method architecture is depicted in Figure 3. An ID generator generates a unique ID for each AV. The generated ID is stored in the respective vehicle’s register, such as $R_{v1}$ and $R_{v2}$ for vehicles one and two, respectively. Similarly, a key for each vehicle is generated by the vehicle center (VC) and stored in a register with *m* locations to accommodate the IDs of *m* vehicles. For example, $R_{k1}$, and $R_{k2}$ hold the keys for vehicles one and two, respectively. These ID and key registers are connected to separate multiplexers. A control unit selects which ID and corresponding key are forwarded to the outputs. Both outputs are then applied to a synchronization buffer and subsequently undergo encryption (denoted as *Encryption 1*).

(1) $${Output}_{\mathrm{Encryption}\;1}=E\left(ID,K\right)$$

where *E* represents the encryption process, ID is the vehicle ID, and *K* is the corresponding key. The control unit manages the registration and encryption operations for each vehicle. When a new vehicle is added to the network, the ID generator creates a new ID for that vehicle. The control unit configures the multiplexer selection signals to enable the new vehicle ID’s path to be forwarded to the VC for registration. When no updates are required and a vehicle needs to encrypt its identification information, the control unit sets the selection signals of the multiplexers to choose the appropriate ID and key, which are then forwarded to the encryption unit. The VC comprises multiple blocks responsible for generating new keys and encrypting a vehicle’s ID with its corresponding key. All generated keys are stored in a dedicated register for future reference. During the registration process, the VC receives the vehicle’s ID and appends it to an ID list managed by the control unit. The control unit determines the storage location for the new ID and instructs the key generator to create a corresponding key. This new key is stored in the key register. All operations are coordinated by the control unit to ensure synchronization. During encryption, the control unit enables the relevant ID and key registers to forward the ID and its key *K* to the encryption unit (denoted as *Encryption 2*).

(2) $${Output}_{\mathrm{Encryption}\;2}=E^{\prime }\left(ID,K\right)$$

where $E^{\prime }$ denotes the second encryption process. Both encrypted outputs, *Encryption 1* and *Encryption 2*, are sent to a synchronization buffer. The buffer output is then used to compare the received encrypted ID information from the vehicle with the encrypted data from the VC.

If the encrypted vehicle ID matches the records saved in the VC, the authentication process confirms the vehicle’s identity and grants access. This comparison is mathematically represented as follows:

(3) $$\mathrm{Match}=\left\{\begin{array}{cc} 1, & \mathrm{if}\;E(ID,K)=E^{\prime }\left(ID,K\right),\\ 0, & \mathrm{otherwise}. \end{array}\right.$$

A successful match (Match=1) indicates that the vehicle’s identity is authenticated, allowing data exchange within the IoV framework. In contrast, a mismatch triggers a denial of access, safeguarding against unauthorized vehicles.

### 6.2. Experimental Results

#### 6.2.1. Communication Overhead

The communication overhead is primarily determined by the size of the encrypted AV’s ID data transmitted to the cloud and the authentication queries. Each of these communications involves vectors made up of binary elements that represent the encrypted information. In our proposed scheme, each encrypted vector contains 8*n binary digits, where n is the ID size, varying from 100 to 800 bits. After encryption, each transmission step results in a total size range from 8 * 100to 8 * 800 to represent the ID size from 100 to 800 bits. This gives our communication overhead a range from 800 bits (100B) to 6400 bits (800B). This consistent message size is used for all transmission steps, including encrypted AV ID data and authentication queries. As a result, the data transmission overhead remains constant and relatively small across scheme, contributing to an efficient and scalable system. These results demonstrate the system’s robustness, with a focus on both the scalability of the encryption process and the efficient communication overhead management.

**Figure 4:**
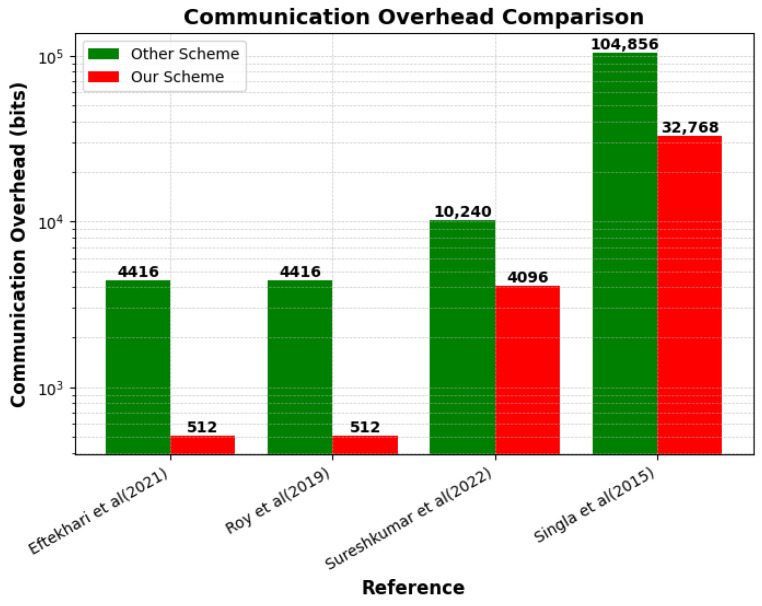
Communication overhead comparison with Refs. [35,37,38,39].

As illustrated in Figure 4, several existing schemes, such as those in [37,38], suffer from significantly higher communication overheads, requiring 4416 bits for an AV ID size of 64 bits. In contrast, our proposed scheme, which handles the submission of authentication queries and the matching of encrypted data to retrieve authentication results, achieves the same tasks with only 512 bits for the same ID size, demonstrating a substantial reduction in overhead. Similarly, while [39] demands 10,240 bits for an ID size of 512 bits, our scheme optimizes this to just 4096 bits for the same ID size, enhancing efficiency without sacrificing performance. The most resource-intensive scheme, [35], requires 104,856 bits for the ID size of 4096 bits, whereas our approach reduces this to 1280 bits, offering a scalable and lightweight solution for secure communication. This analysis underscores the superior ability of our scheme to minimize communication overhead while ensuring both security and efficiency.

#### 6.2.2. Computation Overhead

**Figure 5:**
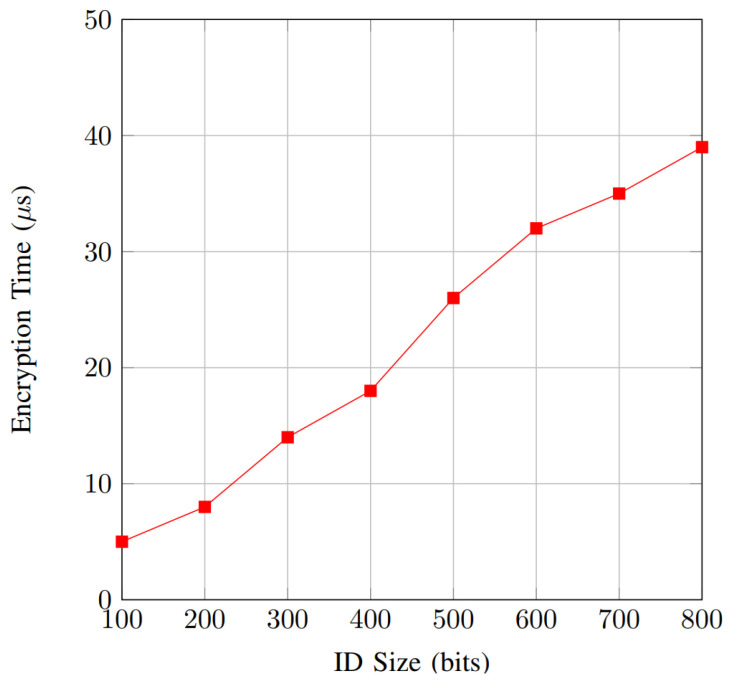
Encryption time.

**Figure 6:**
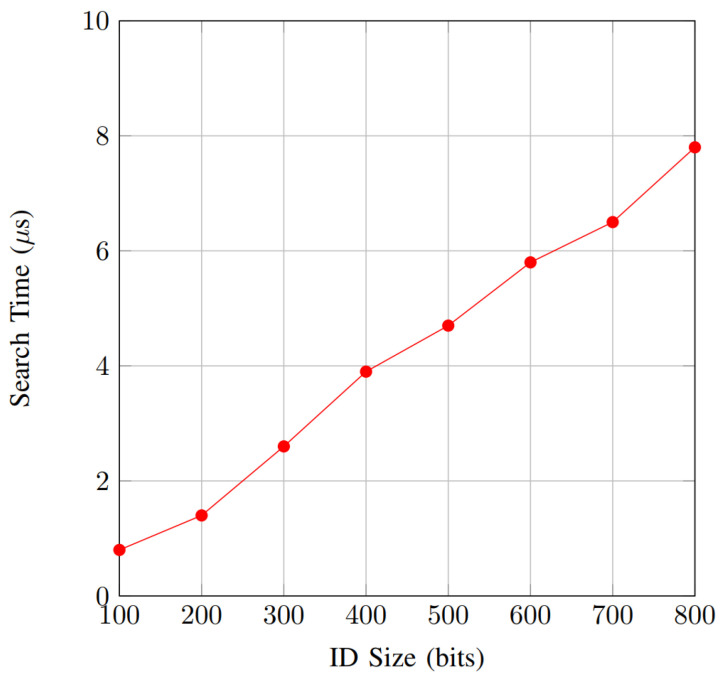
Search time.

The proposed method was successfully implemented on the Nexys A7-100T FPGA, Xilinx, Inc., San Jose, CA, USA which operates at 100 MHz. The encryption time was employed to evaluate the performance of the proposed method, as depicted in Figure 5. The results reveal that the encryption time increases with the ID size (n) growing, albeit with relatively small values. The hardware implementation requires less time than the software, which can be very useful in cases where we need to process a large number of authentication requests. Comparable performance is observed in the search time, illustrated in Figure 6. The overhead in search time does not vary significantly across different ID sizes.

Figure 5 illustrates the relationship between encryption time and ID size for the proposed authentication scheme implemented on the Nexys A7-100T FPGA. The encryption time increases as the ID size grows, demonstrating a non-linear trend. Specifically, the encryption time starts at 5 μs for an ID size of 100 bits and gradually rises to 39 μs when the ID size reaches 800 bits. The initial increase is relatively moderate, but as the ID size exceeds 400 bits, the encryption time escalates more rapidly, reflecting the increased computational complexity required to process larger IDs. This trend highlights the impact of ID size on encryption performance, emphasizing the need to optimize the encryption algorithm for larger IDs to maintain acceptable performance levels in real-time applications.

Figure 6 shows the search time performance as a function of ID size. The results indicate a gradual increase in search time with the growing ID size, but the growth rate is relatively slow compared to the encryption time. Starting at 0.8 μs for an ID size of 100 bits, the search time steadily climbs to 7.8 μs at 800 bits. This linear growth suggests that the search operation’s efficiency is less impacted by ID size than encryption, likely due to optimized search algorithms (its lightweight operations) or the inherent parallelism of the FPGA implementation. The controlled increase in search time demonstrates the scalability of the search process, making it suitable for applications that involve large ID spaces without significantly compromising performance. These observations from the results demonstrate that while both encryption and search times increase with larger ID sizes, the rate and nature of this increase differ, underlining the importance of balancing performance considerations when designing hardware-accelerated, privacy-aware authentication schemes for IoV systems.

**Figure 7:**
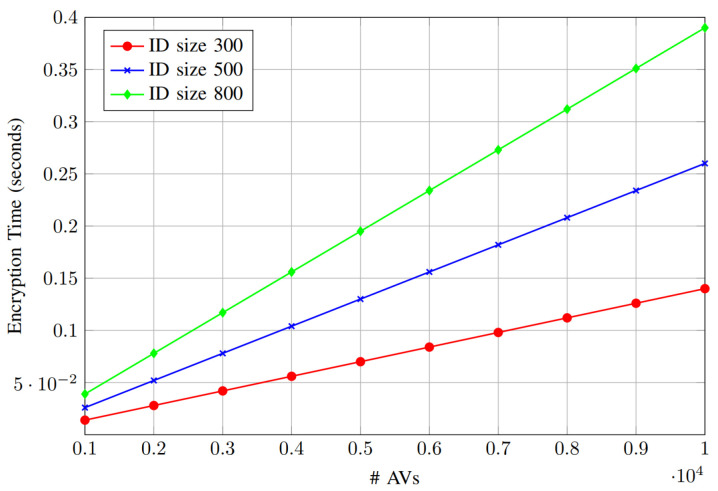
Encryption time vs. number of AVs.

**Figure 8:**
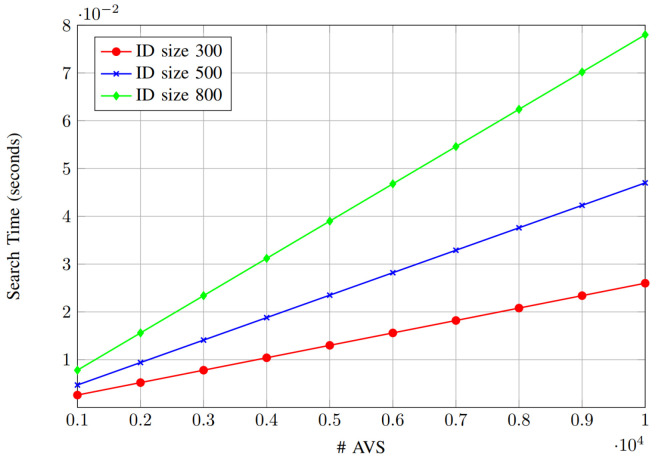
Search time vs. number of AVs.

The results presented in Figure 7 and Figure 8 illustrate the impact of varying ID sizes (security levels different from the AV’s ID will determine the key size) on encryption and search time as the number of AVs increases (1000–10,000 AVs to represent large-scale IoV). As expected, both encryption and search times increase proportionally with the number of AVs, reflecting the linear dependency on AV count. For encryption time (Figure 7), an ID size of 300 results in the lowest encryption overhead, while larger ID sizes of 500 and 800 lead to higher encryption times due to increased computational complexity. Similarly, the search time analysis (Figure 8) shows that, as the ID size increases, the time required to search for a specific ID also rises. Specifically, the search time remains minimal for an ID size of 300, whereas for an ID size of 800, the search process becomes more time-intensive. These results highlight the trade-off between security (through larger ID sizes) and efficiency (lower computational cost), emphasizing the need to balance both aspects for real-time applications.

**Figure 9:**
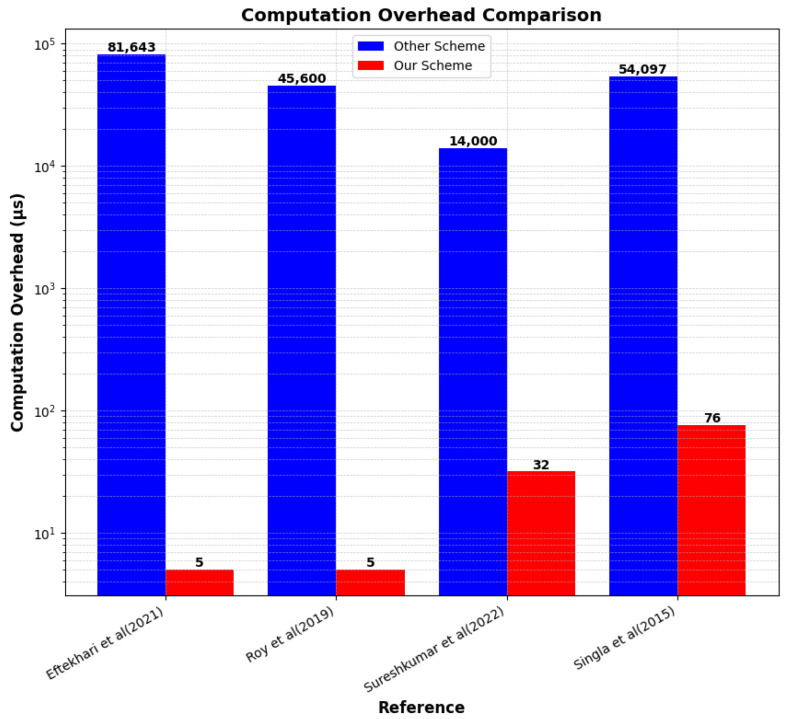
Computational overhead comparison with Refs. [35,37,38,39].

In Figure 9, in contrast to other methods, such as [37,38], which take 81,643 μs and 45,600 μs, respectively, our proposed method significantly lowers the computational burden by accomplishing the same tasks in just 5 μs. This illustrates a remarkable enhancement in computational efficiency. Similarly, while [35,39] require 14,000 μs and 54,097 μs, respectively, our method streamlines computation time to only 32 μs and 76 μs, preserving high performance with minimal overhead.

#### 6.2.3. Hardware Resource Utilization

The proposed method was successfully implemented on the Nexys A7-100T FPGA, with an analysis of the resource utilization presented in Table 2. The implementation utilized 15,400 look-up tables (LUTs) out of the available 63,400, resulting in a 24.3% usage, which reflects the efficient utilization of FPGA logic resources. Additionally, the design required 7100 flip-flops (FFs) out of 126,800 available, accounting for 5.6% utilization and demonstrating that the sequential logic demands were minimal. The block RAM (BRAM) utilization was 22.2%, using 30 out of 135 available blocks and indicating moderate memory requirements for the proposed architecture. The design also employed 60 digital signal processing (DSP) slices, which represent 25% of the available 240 slices, showing that the method efficiently handles arithmetic operations. Finally, the implementation used 50 out of 200 available IO pins, accounting for 25% of the total, which indicates that the design has reasonable input/output interfacing demands. Overall, the resource utilization metrics suggest that the proposed authentication scheme is resource-efficient and scalable, making it well suited for real-time applications on FPGA platforms.

Table 3 shows the comparative analysis of resource utilization across various schemes, focusing on the proposed scheme’s efficiency under consideration. Our scheme exhibits the minimal utilization of lookup tables (LUTs), necessitating merely 15,400 and thereby significantly surpassing the performance of alternative methodologies. In contrast, the work referenced in [40] utilizing HBA, XTEA, and present schemes, necessitate 419,000, 433,200, and 428,500 LUTs, respectively. Additionally, our scheme uses only 9340 slices, much lower than other schemes, which require large numbers of slices. The MA utilizing HBA accounts for 223,000 slices, while the MA employing XTEA and Present utilize 225,500 and 223,200 slices, respectively. Furthermore, our scheme consumes only 7100 LUT-flip-flops (LUT-FFs), in stark contrast to the other methodologies, which incur substantially higher utilizations—specifically, MA utilizes HBA accounts for 242,000 LUT-FFs, while MA employing XTEA and Present utilize 260,000 and 256,000 LUT-FFs, respectively. In summary, our scheme is characterized by superior efficiency in terms of resource utilization, providing an optimal equilibrium between LUTs, slices, and LUT-FFs and thereby establishing itself as a more advantageous option than the alternatives.

#### 6.2.4. PUF Performance Analysis

**Figure 10:**
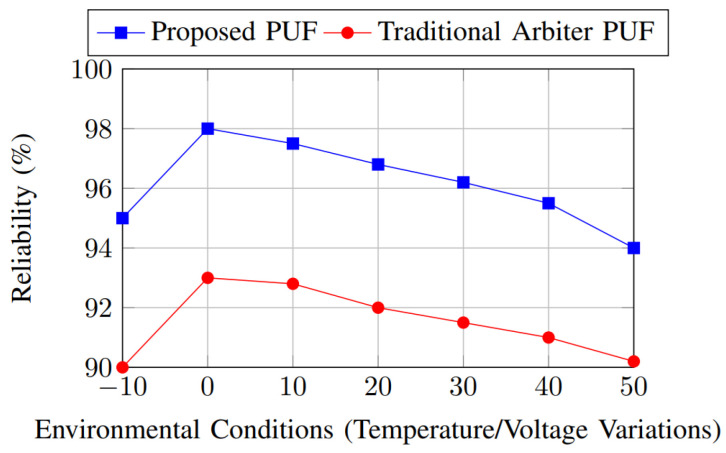
Reliability comparison.

The reliability of the proposed PUF consistently outperforms the traditional arbiter PUF under environmental variations, as shown in Figure 10. The proposed PUF maintains reliability above 94%, while the traditional arbiter PUF drops to 90% at extreme conditions, indicating better resilience to environmental stress for the proposed design. This demonstrates that the proposed PUF is resilient to environmental variations, making it suitable for practical applications.

**Figure 11:**
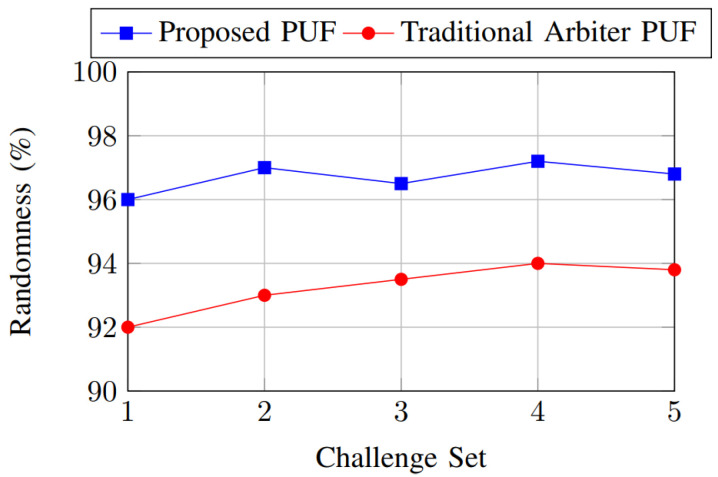
Randomness comparison.

Randomness was evaluated by analyzing the distribution of responses to a set of challenges, as shown in Figure 11. The randomness evaluation shows that the proposed PUF achieves consistently higher randomness, exceeding 96% across all challenge sets. In contrast, the traditional arbiter PUF exhibits lower randomness, peaking at 94%. This demonstrates the enhanced unpredictability of the proposed PUF’s response generation.

**Figure 12:**
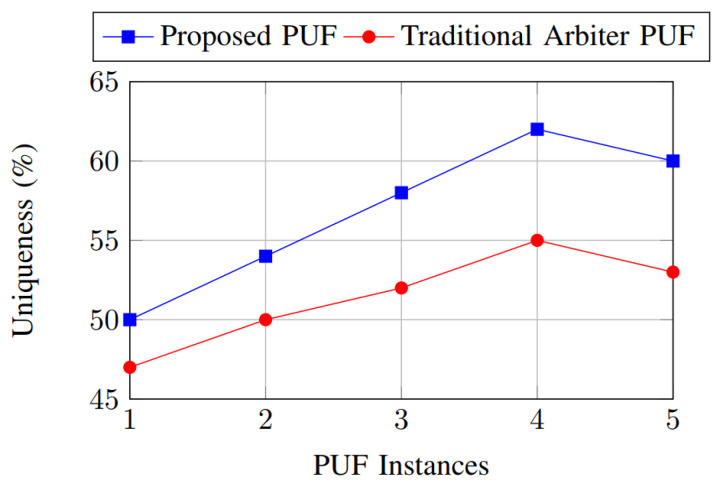
Uniqueness comparison.

The uniqueness metric measures the ability of the PUF to generate distinct responses for different instances. As shown in Figure 12, the uniqueness metric highlights the ability of the proposed PUF to generate distinct responses for different instances, with values reaching up to 62%. The traditional arbiter PUF shows lower uniqueness, with a maximum of 55%. This confirms that the proposed PUF provides better device-level differentiation, ensuring robust authentication.

To further reinforce the security advantages of the proposed PUF, we conducted a quantitative security evaluation, including attack resistance and key entropy analysis. The attack resistance was assessed against machine learning (ML)-based modeling attacks using a dataset of challenge–response pairs (CRPs). The proposed PUF demonstrated superior robustness, requiring more than $10^{6}$ CRPs for an ML attack to achieve an accuracy above 50%, whereas the traditional arbiter PUF was compromised with only $10^{4}$ CRPs. This highlights the enhanced resilience of the proposed design to ML-based threats. Furthermore, key entropy analysis was performed to measure the unpredictability of responses. The Shannon entropy of the proposed PUF was calculated as 0.997, compared to 0.985 for the traditional arbiter PUF, indicating a higher level of randomness. The min entropy, which represents the worst-case predictability, was also higher for the proposed PUF (0.92) than the arbiter PUF (0.88), confirming its superior resistance to entropy-based attacks. Additionally, bit aliasing analysis revealed that the proposed PUF exhibited a nearly uniform bit distribution (49.8% ‘1’s and 50.2% ‘0’s), reducing bias and ensuring balanced response generation.

The scalability of the proposed PUF design was evaluated by analyzing its adaptability to different hardware platforms. As the number of CRPs increases, the resource utilization scales efficiently, with LUT and FF consumption growing linearly. This ensures that the design remains viable even for high-security applications requiring a large CRP space. Our implementation demonstrates reasonable DSP and BRAM usage, making it suitable for high-performance FPGAs and resource-constrained platforms such as low-power IoT devices.

## 7. Conclusions

The Internet of Vehicles (IoV) serves as a fundamental technology connecting smart cities and automobiles to enhance transportation safety and efficiency. This integrated network enables wireless communication among vehicles, facilitating the exchange of crucial traffic data. However, this progress raises privacy concerns for individual users. This paper introduces an innovative privacy-preserving authentication scheme utilizing the k-nearest neighbor (KNN) encryption algorithm. The proposed approach aims to surpass the limitations of current solutions by introducing a multi-multi searching property for authenticating AVs in the IoV era. Our scheme is designed to safeguard against internal and external threats, ensuring the integrity of the IoV ecosystem and preserving user privacy. To efficiently process the substantial amount of information required for authentication, our technique employs hardware acceleration, utilizing a Physically Unclonable Function (PUF) to generate unique keys randomly and FPGA boards. Our privacy and security analysis indicates that our proposed method successfully achieves the required design objectives. Furthermore, a performance evaluation revealed that our system imposes minimal communication and computational overheads and consumes fewer resources than existing schemes.

## Figures and Tables

**Table 1:** Main notations.

Symbol	Description
PUF	Physical unclonable function.
KNN	k-nearest neighbor encryption technique.
AV	Autonomous vehicle.
TM	Transport management entity.
AS	Authentication server.
TTP	Trusted third party.
*n*	Size of identification vectors (e.g., AV ID data).
*B*	A random binary vector of size *n* used as part of the key generation for scheme members.
P1,P2	Invertible random matrices of size n×n used in the key generation of the TTP.
M1,M2,⋯,M8	Invertible random matrices of size n×n that form part of the TTP secret key.
TTPK	TTP secret key, defined as {B,P1,P2,M1,⋯,M8}.
*A*	An invertible random matrix in $R^{n\times n}$ used to generate the authentication server secret key (ASK).
$C_{i},\;D_{i},E_{i},\;F_{i}$	Random matrices in $R^{n\times n}$ used in TM key generation, satisfying $C_{i}+D_{i}=P_{1}^{-1}$ and $E_{i}+F_{i}=P_{2}^{-1}$.
$TMK_{i}$	Secret key for transport management entity $TM_{i}$
$H_{x},\;J_{x},\;K_{x},\;N_{x}$	Random matrices in $R^{n\times n}$ used in AV key generation, with $H_{x}+J_{x}=P_{1}$ and $K_{x}+N_{x}=P_{2}$.
$AVK_{x}$	Secret key for an autonomous vehicle.
$q_{i}$	Identification data vector of an AV as managed by $TM_{i}$.
$q_{i}^{\prime },\;q_{i}^{\prime \prime }$	Two column vectors obtained by splitting $q_{i}$ using the binary vector *B*.
$S_{ix}$	Ciphertext (encrypted index) generated by $TM_{i}$ for an $AV_{x}$.
$U_{x}$	Original identification vector of $AV_{x}$.
$u_{x}^{\prime },\;u_{x}^{\prime \prime }$	Two vectors obtained by splitting $U_{x}$ using *B*.
$CT_{x}$	Trapdoor (encrypted authentication query) generated by $AV_{x}$.
$S_{ix}^{\prime }$	Modified ciphertext obtained by applying $A^{-1}$ to $S_{ix}$, used in the matching process.
*	Dot product operation used in matching the encrypted data.

**Table 2:** Resource utilization on Nexys A7-100T FPGA.

Resource	Available	Used	Utilization (%)
LUTs	63,400	15,400	24.3%
FFs	126,800	7100	5.6%
BRAM	135	30	22.2%
DSP Slices	240	60	25%
IO Pins	200	50	25%

**Table 3:** Comparison of resource utilization.

Resources	Our Scheme	MA Using HBA [40]	MA Using XTEA [40]	MA Using Present [40]
LUT	15,400	419,000	433,200	428,500
Slices	9340	223,000	225,500	223,200
LUT-FF	7100	242,000	260,000	256,000

## Data Availability

Data are contained within the article.
